# Biogeography of the *Phalaenopsis amabilis* species complex inferred from nuclear and plastid DNAs

**DOI:** 10.1186/s12870-015-0560-z

**Published:** 2015-08-16

**Authors:** Chi-Chu Tsai, Chang-Hung Chou, Hao-Ven Wang, Ya-Zhu Ko, Tzen-Yuh Chiang, Yu-Chung Chiang

**Affiliations:** Crop Improvement Division, Kaohsiung District Agricultural Improvement Station, Pingtung, 900 Taiwan; Graduate Institute of Biotechnology, National Pingtung University of Science and Technology, Pingtung, 912 Taiwan; Department of Biological Sciences, National Sun Yat-sen University, Kaohsiung, 804 Taiwan; Research Center for Biodiversity, China Medical University, Taichung, 404 Taiwan; Department of Life Science, National Cheng Kung University, Tainan, 701 Taiwan; Department of Biomedical Science and Environment Biology, Kaohsiung Medical University, Kaohsiung, 807 Taiwan

**Keywords:** Biogeography, Demographic dynamics, *Phalaenopsis amabilis*, Phylogeny, Species complex, Vicariance

## Abstract

**Background:**

*Phalaenopsis* is one of the important commercial orchids in the world. Members of the *P. amabilis* species complex represent invaluable germplasm for the breeding program. However, the phylogeny of the *P. amabilis* species complex is still uncertain. The *Phalaenopsis amabilis* species complex (Orchidaceae) consists of subspecies *amabilis*, *moluccana*, and *rosenstromii* of *P. amabilis*, as well as *P. aphrodite* ssp. *aphrodite*, *P. ap.* ssp. *formosana*, and *P. sanderiana*. The aims of this study were to reconstruct the phylogeny and biogeographcial patterns of the species complex using Neighbor Joining (NJ), Maxinum Parsimony (MP), Bayesian Evolutionary Analysis Sampling Trees (BEAST) and Reconstruct Ancestral State in Phylogenies (RASP) analyses based on sequences of internal transcribed spacers 1 and 2 from the nuclear ribosomal DNA and the *trn*H-*psb*A spacer from the plastid DNA.

**Results:**

A pattern of vicariance, dispersal, and vicariance + dispersal among disjunctly distributed taxa was uncovered based on RASP analysis. Although two subspecies of *P. aphrodite* could not be differentiated from each other in dispersal state, they were distinct from *P. amabilis* and *P. sanderiana*. Within *P. amabilis*, three subspecies were separated phylogenetically, in agreement with the vicariance or vicariance + dispersal scenario, with geographic subdivision along Huxley’s, Wallace’s and Lydekker’s Lines. Molecular dating revealed such subdivisions among taxa of *P. amabilis* complex dating back to the late Pleistocene. Population-dynamic analyses using a Bayesian skyline plot suggested that the species complex experienced an *in situ* range expansion and population concentration during the late Last Glacial Maximum (LGM).

**Conclusions:**

Taxa of the *P. amabilis* complex with disjunct distributions were differentiated due to vicariance or vicariance + dispersal, with events likely occurring in the late Pleistocene. Demographic growth associated with the climatic oscillations in the Würm glacial period followed the species splits. Nevertheless, a subsequent population slowdown occurred in the late LGM due to extinction of regional populations. The reduction of suitable habitats resulted in geographic fragmenttation of the remaining taxa.

**Electronic supplementary material:**

The online version of this article (doi:10.1186/s12870-015-0560-z) contains supplementary material, which is available to authorized users.

## Background

**Fig. 1:**
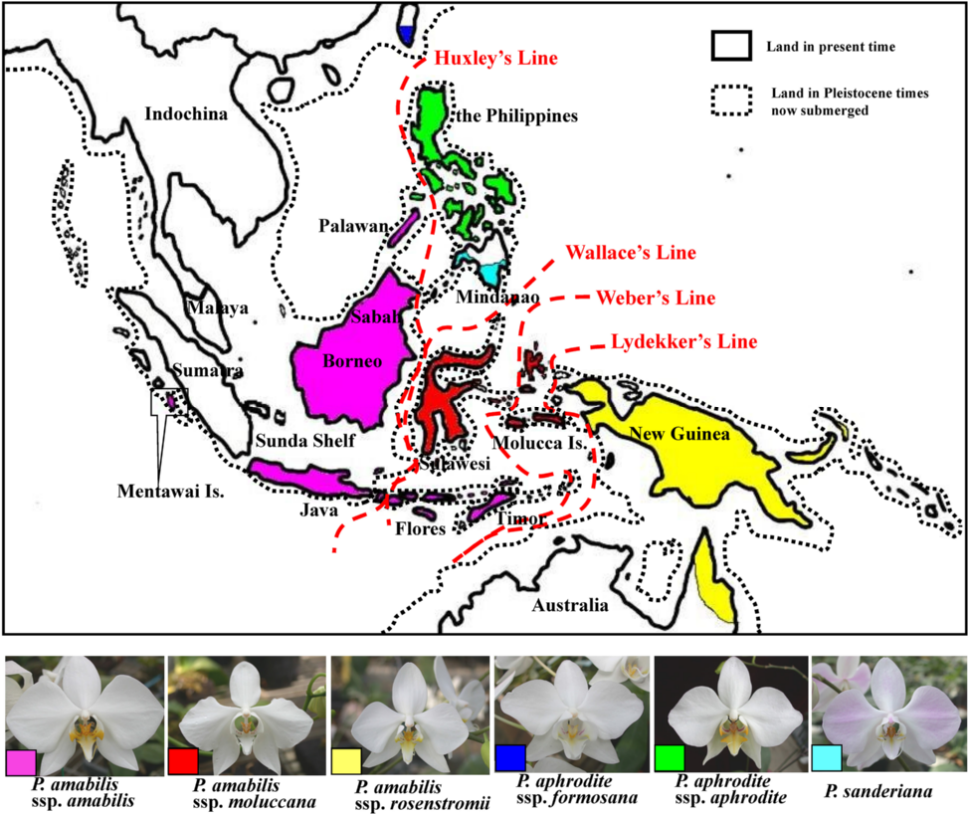
Geographical distribution of six species/subspecies of the *Phalaenopsis amabilis* species complex and Southeast Asia landmasses between the Pleistocene and the present. In Pleistocene times, Indochina, Malaya, Sumatra, Java, Borneo, and the Philippines were interconnected and were separated from Sulawesi by the Makassar Strait. Four phylogeographic break lines were shown in red dashed lines (modified from [6]) and distribution region of six species/subspecies of the *Phalaenopsis amabilis* species complex drawed by different color in the map. Images for six species/subspecies of the *Phalaenopsis amabilis* species complex were photographed by CC Tsai (the first author)

According to the historical geology of Southeast Asia, most Philippine islands are relatively young, originating about five million years ago (Mya) [1, 2]. The older islands of the Philippines, including Palawan, Mindoro and Zamboanga, are located on the edges of the Eurasian Plate and may have been moving away from the main landmass since the early Miocene (approximately 30 Mya). Until approximately 5–10 Mya, the crust of the older plate was a part of Borneo [3–5]. The Malay Peninsula, Borneo, Sumatra and Java together comprise the Sunda Shelf. When sea levels were low during the glacial maxima, the Malay Peninsula, Borneo, Sumatra, Java, Bali and the Philippines were interconnected via land bridges, making species migrations possible [6]. In addition, western Sulawesi had been a part of the Sunda Shelf in ancient times but slid away during the Palaeocene (approximately 50 Mya). The formation of the deep Makassar Strait divided western Sulawesi from the Sunda Shelf, preventing further dispersal of Bornean species to Sulawesi. This history geology accounts for the high degree of endemism among the fauna and flora of Sulawesi [7] (Fig. 1).

The *P. amabilis* species complex includes *P. amabilis* ssp. *amabilis*, *P. amabilis* ssp. *moluccana*, *P. amabilis* ssp. *rosenstromii*, *P. aphrodite* ssp. *aphrodite*, *P. aphrodite* ssp. *formosana* and *P. sanderiana*. These species and their subspecies were placed in the section *Phalaenopsis* [8, 9]. The species complex spans a broad geographic range, including southeastern Taiwan, the Philippines, Borneo, Sumatra (Mentawai Is.), Java, Sulawesi, Molucca Is., New Guinea, and northeastern Australia (Queensland) (Fig. 1), and has a complicated biogeography because these regions border two palaeocontinents [10]. These regions have long attracted the attention of biogeographers, and several phylogeographical breaks have been introduced (Fig. 1) [6, 7, 11]. Species/subspecies of the species complex shared most morphological characters, except for lip-midlobes and calli. The distributional patterns coincided with the geographic isolations via historical phylogeographic breaks between Borneo + Palawan and the Philippines (Huxley’s Line), between Borneo and Sulawesi (Wallace’s Line), between Sulawesi and Molucca Is. (Weber’s Line), and between Sulawesi and New Guinea + Australia (Lydekker’s Line) [6, 7, 11, 12].

*Phalaenopsis amabilis* ssp*. amabilis* is widespread, extending from the southern Philippines (Palawan) to Borneo, Sumatra and Java. Another subspecies, *P. amabilis* ssp. *moluccana*, is strictly distributed in the Molucca Islands (Seram, Buru) and Sulawesi. There is a phylogeographic break (Wallace’s Line) between these two subspecies (Fig. 1) [6, 11]. *Phalaenopsis amabilis* ssp. *moluccana* is easily recognised by the shape of the midlobe of the lip, which is linear-oblong with a slight dilation toward the base instead of being cruciform [8] (Table 1). *P. amabilis* ssp. *rosenstromii* is distributed in New Guinea (Kaiser Wilhelms Land), New Ireland (Neumecklenburg) and Australia (northeastern Queensland). This subspecies differs from *P. amabilis* ssp. *amabilis* and *P. amabilis* ssp. *moluccana* in having a lip with a triangular midlobe, as in *P. aphrodite* and *P. sanderiana* (Table 1). *Phalaenopsis amabilis* ssp. *moluccana* and *P. amabilis* ssp. *rosenstromii* are separated by a phylogeographic break at the Lydekker’s Line [12].

**Table 1:** Differentiated characters among six species/subspecies of the *Phalaenopsis amabilis* species complex

Taxa	Morphological characters				
	Floral color	Lip color	Leaves with anthocyanin pigment	Teeth no. of posterior edge in calli	Midlobe shape of labium
*P. amabilis* ssp. *amabilis*	White	Base of lip marked with yellow and red	No	Two	Cruciform
*P. amabilis* ssp. *moluccana*	White	Base of lip marked with yellow, white	No	Two	Linear-oblong with a slight dilation toward the base
*P. amabilis* ssp. *rosenstromii*	White	Base of lip marked with yellow	No	Two	The shorter, narrowly triangular midlobe of the lip, with inconspicuous teeth at the base
*P. aphrodite* ssp. *aphrodite*	White	Base of lip marked with yellow and red	Various	Four^a^	Triangular-hastate midlobe
*P. aphrodite* ssp. *formosana*	White	Base of lip marked with yellow	No	Four^a^	Triangular-hastate midlobe
*P. sanderiana*	White, pink	Base of lip marked with yellow and red	Yes	Four^b^	Triangular-hastate midlobe

^a^outer two teeth longer than the inner two

^b^inner two teeth longer than the outer two

Given high resemblance between *P. aphrodite* and *P. amabilis*, *P. aphrodite* was ever once placed under *P. amabilis*, i.e., *P. amabilis* var. *aphrodite* [8, 9]. In contrast, Sweet [13] recognised it as a distinct species based on characteristics of the callus and midlobe shape, and the geographical subdivision by the Lydekker’s Line [12]. *P. aphrodite* ssp. *aphrodite* is distributed throughout the Philippines with exceptions of Mindanao and Palawan [13]. Its calli have a posterior edge dividing into four teeth, unlike the two-toothed calli of *P. amabilis* [9] (Fig. 1). Another subspecies, *P. aphrodite* ssp. *formosana*, is distributed only in southeastern Taiwan and can be distinguished from *P. aphrodite* ssp. *aphrodite* by its apple-green leaves with no trace of anthocyanin pigments, somewhat smaller flowers, and many-branched panicles [9] (Table 1).

Another phyloegnetically related species [14], *P. sanderiana*, has a restricted distribution in the southern Philippines (including Mindanao Island, Igat Island and Balut and Sarangani Islands) [15]. Its callus structure shows four teeth, with the inner teeth longer than the outer ones. The four teeth of *P. aphrodite*, in contrast, are subequal, with the two outer teeth only slightly longer than the inner ones [9] (Fig. 1). The distributions of *P. sanderiana* and *P. amabilis* ssp. *amabilis* are divided by Huxley’s Line, and those of *P. sanderiana* and *P. amabilis* ssp. *moluccana* are divided by Wallace’s Line [11].

It is known that phylogeographic patterns are determined by the interplay between dispersal and vicariance [16]. Vicariance arises from changes in the earth’s surface from changes in sea level or the separation of similar environments by climatic, geological or other changes in intervening regions, resulting in the fragmentation of continuously distributed taxa [17]. Dipersal across isolating barriers, which can disrupt the preexisting vicaricance patterns, might also predominate in the oceanic island biogeography [18]. This species complex is a good tool by which to study biogeography because this *P. amabilis* complex is distributed throughout the region between Southeast Asia and Australia, where several phylogeographic break lines have been identified. Here, based on the distribution patterns of the taxa in the species complex across these geographical regions, if vicariance exclusively determined the species split, one would expect reciprocal monophyly of *P. aphordite*, *P. amabilis*, and *P. sanderiana,* which were divided by Huxley’s and Wallace’s Lines. Affinity among *P. amabilis* ssp. *amabilis*, ssp. *moluccana*, and ssp. *rosenstromii* separated by Wallace’s and Weber’s Lines remains testable. In other word, Christenson’s [9] taxonomy is served as the biogeographical hypothesis.

In this study, DNA fragments of the internal transcribed spacer (ITS) of nuclear ribosomal DNA and the *trn*H-*psb*A intergenic spacer of plastid DNA were employed. Several questions were addressed. 1) Is monophyly of the species of the complex supported? 2) Is the geographic disjunction in this species complex due to vicariance or long-distance dispersal? 3) What is the demographic history of *P. amabilis* complex? 4) What is the extent of gene flow between species?

## Results

### Sequence characteristics, variation and haplotype diversity

For all accessions of the *P. amabilis* species complex (Table 2), the ITS1 sequence lengths ranged from 228 to 233 base pairs (bp); the 5.8S-rDNA sequences were 163 bp, and the ITS2 sequences ranged from 256 to 258 bp (GenBank Nos. AY391515-53; http://dx.doi.org/10.5061/dryad.f8j12). There were no nucleotide substitutions or indels in the 5.8S rDNA of the *P. amabilis* complex. Both variable and potential parsimony-informative sites in the ITS1 region across the species complex are higher than those of ITS2 region. The ITS1 and ITS2 sequences of the 39 accessions of the *P. amabilis* complex were combined and aligned, yielding 494 characters. Eight gap sites, 13 variable sites and 11 potential parsimony-informative sites were found in the aligned matrix (Table 3).

**Table 2:** A list of the 39 accessions of the *Phalaenopsis amabilis* species complex, namely *P. amabilis*, *P. aphrodite*, and *P. sanderiana*, and their different geographical distributions

Taxa and systematic classification^a^	Distribution	Source^b^/Voucher^c^
*P. amabilis* ssp. *amabilis* ‘Java’	Bantam, Java, Indonesia	KDAIS-kc66/Tsai C.C. 1066
*P. amabilis* ssp. *amabilis* ‘Java’	Bantam, Java, Indonesia	KDAIS-kc96/Tsai C.C. 1096
*P. amabilis* ssp. *amabilis* ‘Java’	Bantam, Java, Indonesia	KDAIS-kc97/Tsai C.C. 1097
*P. amabilis* ssp. *amabilis* ‘Mentawai Is.’	Mentawai Is., Sumatra, Indonesia	KDAIS-kc238/Tsai C.C. 1238
*P. amabilis* ssp. *amabilis* ‘Mentawai Is.’	Mentawai Is., Sumatra, Indonesia	KDAIS-kc239/Tsai C.C. 1239
*P. amabilis* ssp. *amabilis* ‘Mentawai Is.’	Mentawai Is., Sumatra, Indonesia	KDAIS-kc240/Tsai C.C. 1240
*P. amabilis* ssp. *amabilis* ‘Palawan’	Brooks Point, Palawan, the Philippines	KDAIS-kc91/Tsai C.C. 1091
*P. amabilis* ssp. *amabilis* ‘Palawan’	Brooks Point, Palawan, the Philippines	KDAIS-kc92/Tsai C.C. 1092
*P. amabilis* ssp. *amabilis* ‘Palawan’	Brooks Point, Palawan, the Philippines	KDAIS-kc93/Tsai C.C. 1093
*P. amabilis* ssp. *amabilis* ‘Sabah’	Sabah, Indonesia	KDAIS-kc327/Tsai C.C. 1327
*P. amabilis* ssp. *amabilis* ‘Sabah’	Sabah, Indonesia	KDAIS-kc342/Tsai C.C. 1342
*P. amabilis* ssp. *amabilis* ‘Sabah’	Sabah, Indonesia	KDAIS-kc444/Tsai C.C. 1444
*P. amabilis* ssp. *amabilis* ‘Timor’	East Timor	KDAIS-kc254/Tsai C.C. 1254
*P. amabilis* ssp. *amabilis* ‘Timor’	East Timor	KDAIS-kc343/Tsai C.C. 1343
*P. amabilis* ssp. *moluccana*	Celebes, Molucca Is., Indonesia	KDAIS-kc248/Tsai C.C. 1248
*P. amabilis* ssp. *moluccana*	Celebes, Molucca Is., Indonesia	KDAIS-kc249/Tsai C.C. 1249
*P. amabilis* ssp. *moluccana*	Celebes, Molucca Is., Indonesia	KDAIS-kc319/Tsai C.C. 1319
*P. amabilis* ssp. *rosenstromii*	Kaiser Wilhelms, New Guinea	KDAIS-kc94/Tsai C.C. 1094
*P. amabilis* ssp. *rosenstromii*	Kaiser Wilhelms, New Guinea	KDAIS-kc95/Tsai C.C. 1095
*P. amabilis* ssp. *rosenstromii*	Kaiser Wilhelms, New Guinea	KDAIS-kc260/Tsai C.C. 1260
*P. amabilis* ssp. *rosenstromii*	Kaiser Wilhelms, New Guinea	KDAIS-kc329/Tsai C.C. 1329
*P. aphrodite* ssp. *aphrodite*	Mindanao, the Philippines	KDAIS-kc172/Tsai C.C. 1172
*P. aphrodite* ssp. *aphrodite*	Mindanao, the Philippines	KDAIS-kc173/Tsai C.C. 1173
*P. aphrodite* ssp. *aphrodite*	Mindanao, the Philippines	KDAIS-kc174/Tsai C.C. 1174
*P. aphrodite* ssp. *aphrodite*	Manila, Luzon, the Philippines	KDAIS-kc419/Tsai C.C. 1419
*P. aphrodite* ssp. *aphrodite*	Manila, Luzon, the Philippines	KDAIS-kc420/Tsai C.C. 1420
*P. aphrodite* ssp. *aphrodite*	Manila, Luzon, the Philippines	KDAIS-kc421/Tsai C.C. 1421
*P. aphrodite* ssp. *aphrodite* ‘Fuga Is.’	Fuga Is., the Philippines	KDAIS-kc171/Tsai C.C. 1171
*P. aphrodite* ssp. *aphrodite* ‘Calayan Is.’	Calayan Is., the Philippines	KDAIS-kc169/Tsai C.C. 1169
*P. aphrodite* ssp. *aphrodite* ‘Calayan Is.’	Calayan Is., the Philippines	KDAIS-kc181/Tsai C.C. 1181
*P. aphrodite* ssp. *formosana*	Southern Taiwan	KDAIS-kc179/Tsai C.C. 1179
*P. aphrodite* ssp. *formosana*	Southern Taiwan	KDAIS-kc180/Tsai C.C. 1180
*P. aphrodite* ssp. *formosana*	Southern Taiwan	KDAIS-kc198/Tsai C.C. 1198
*P. aphrodite* ssp. *formosana*	Southern Taiwan	KDAIS-kc199/Tsai C.C. 1199
*P. aphrodite* ssp. *formosana*	Southern Taiwan	KDAIS-kc202/Tsai C.C. 1202
*P. aphrodite* ssp. *formosana*	Southern Taiwan	KDAIS-kc253/Tsai C.C. 1253
*P. sanderiana*	Southern Mindanao, the Philippines	KDAIS-kc35/Tsai C.C. 1035
*P. sanderiana*	Southern Mindanao, the Philippines	KDAIS-kc175/Tsai C.C. 1175
*P. sanderiana*	Southern Mindanao, the Philippines	KDAIS-kc176/Tsai C.C. 1176

^a^The classification of *Phalaenopsis* is based on Christenson (2001)

^b^Kaohsiung District Agricultural Improvement Station

^c^Voucher specimens were deposited at the herbarium of National Museum of Natural Science, Taiwan (TNM)

**Table 3:** Comparisons of sequence divergence and phylogenetic information from variable sites among ITS region of nrDNA and four DNA fragments of chloroplast DNA in *Phalaenopsis amabilis* species complex

DNA region	Length (average)	Average GC content (%)	Nucleotide diversity^a^ (π)	Number of variable sites	Number of informative sites^b^	Number of indels	Number of informative indels^c^	Tajima’ *D* ^d^
ITS1	228-233	77.1	0.0078	8	7	6	1	−0.2170
5.8S rDNA	163	65	0	0	0	0	0	-
ITS2	255-257	77.1	0.0039	5	4	1	0	−0.4274
*trn*H-*psb*A spacer	918-946	33.3-33.9	0.0035	11	8	5	4	−1.6542

^a^Average of percentage pairwise sequence divergences estimated using the Jukes-Cantor model. The same species were sequenced for these six regions

^b^At a phylogenetically informative sites, a nucleotide substitution is shared by two or more species

^c^At a phylogenetically informative indels, an indel is shared by two or more species

^d^Tajima’s test of neutrality for nucleotide substitution. *Significant, *p* < 0.05

Sequence lengths for the *trn*H-*psb*A spacer ranged from 918 to 946 bp (GenBank Nos. FJ460366-407; http://dx.doi.org/10.5061/dryad.f8j12). The sequences of the 39 accessions of the *P. amabilis* complex were aligned, with a sequence length of 908 characters (http://dx.doi.org/10.5061/dryad.f8j12). Five gap sites, 11 variable sites and eight potential parsimony-informative sites were found in the aligned matrix (Table 3). These sites included three long indels, resulting from differences in the copy number of several types of repeat sequences. Two copies of CAATATCTTGTTCTTAGA (extra copy at position 161–178 of the aligned sequences) were found in the two accessions of *P. amabilis* ssp. *amabilis* from Timor, and two copies of TGAAATGAA (extra copy at position 275–283 of the aligned sequences) were found in the four accessions of *P. amabilis* ssp. *rosenstromii*. Most samples from the complex had only one copy of this sequence, but it was absent in *P. amabilis* ssp. *amabilis*-Sabah-kc-342. Two copies of TGAATGAT (extra copy at position 298–306 of the aligned sequences) were found in *P. amabilis* ssp*. amabilis*-Java-kc-97. Most taxa of the complex possessed a single copy of this sequence. Five accessions from *P. aphrodite* ssp. *aphrodite*, including populations from Mindanao and Fuga Island and one accession from the Calayan Island population, did not have this sequence (Additional file 1: Table S1). Of five indels across the sequence alignment of the *trn*H-*psb*A spacer, four are informative (Table 3).

Estimates of nucleotide diversity, haplotype, and haplotype diversity for different species/subspecies of the *P. amabilis* complex were calculated (Table 4). Total nucleotide diversity (π) in ITS1, 5.8 s rDNA, ITS2, and *trn*H-*psb*A spacer of *P. amabilis* complex was 0.0078, 0, and 0.0039, 0.895, and 0.0035, respectively (Table 3). In the ITS region, nucleotide diversity in the *P. amabilis* (π = 0.0054) was higher than that in *P. aphrodite* and *P. sanderiana*, while similar results were found in haplotype diversity (*P. amabilis*: *h* = 0.857; *P. aphrodite*: *h* = 0.222; *P. sanderiana*: *h* = 0.667) (Table 4). In *trn*H-*psb*A region, nucleotide diversity in the *P. sanderiana* (π = 0.0029) was higher than that in *P. amabilis* and *P. aphrodite*, while haplotype diversity in the *P. amabilis* (*h* = 0.758) was the hightest among taxa (Table 4). In short, the nucleotide diversity of both ITS1 and ITS 2 regions across the species complex is higher than that of the *trn*H-*psb*A spacer.

**Table 4:** Species names, number of haplotypes, haplotype diversity (*h*), nucleotide diversity (*π*) for each species/subspecies of the *Phalaenopsis amabilis* species complex based on ITS1 + ITS2 region of nrDNA and the *trn*H-*psb*A spacer of chloroplast DNA

Species	No. of accessions	The ITS region/the *trn*H-*psb*A spacer				
		No. of haplotypes	Polymorphic sites	Parsimony informative sites	*h*	π
*P. amabilis* ssp. *amabilis*	14	6/5	8/4	6/2	0.857/0.758	0.0054/0.0012
*P. amabilis* ssp. *moluccana*	3	1/1	0/0	0/0	0.000/0.000	0.0000/0.0000
*P. amabilis* ssp. *rosenstromii*	4	1/2	0/1	0/0	0.000/0.500	0.0000/0.0005
*P. aphrodite* ssp. *aphrodite*	9	2/1	1/0	0/0	0.222/0.000	0.0005/0.0000
*P. aphrodite* ssp. *formosana*	6	1/2	0/1	0/1	0.000/0.533	0.0000/0.0006
*P. sanderiana*	3	2/2	2/4	0/0	0.667/0.667	0.0028/0.0029

### Phylogenetic reconstruction

**Fig. 2:**
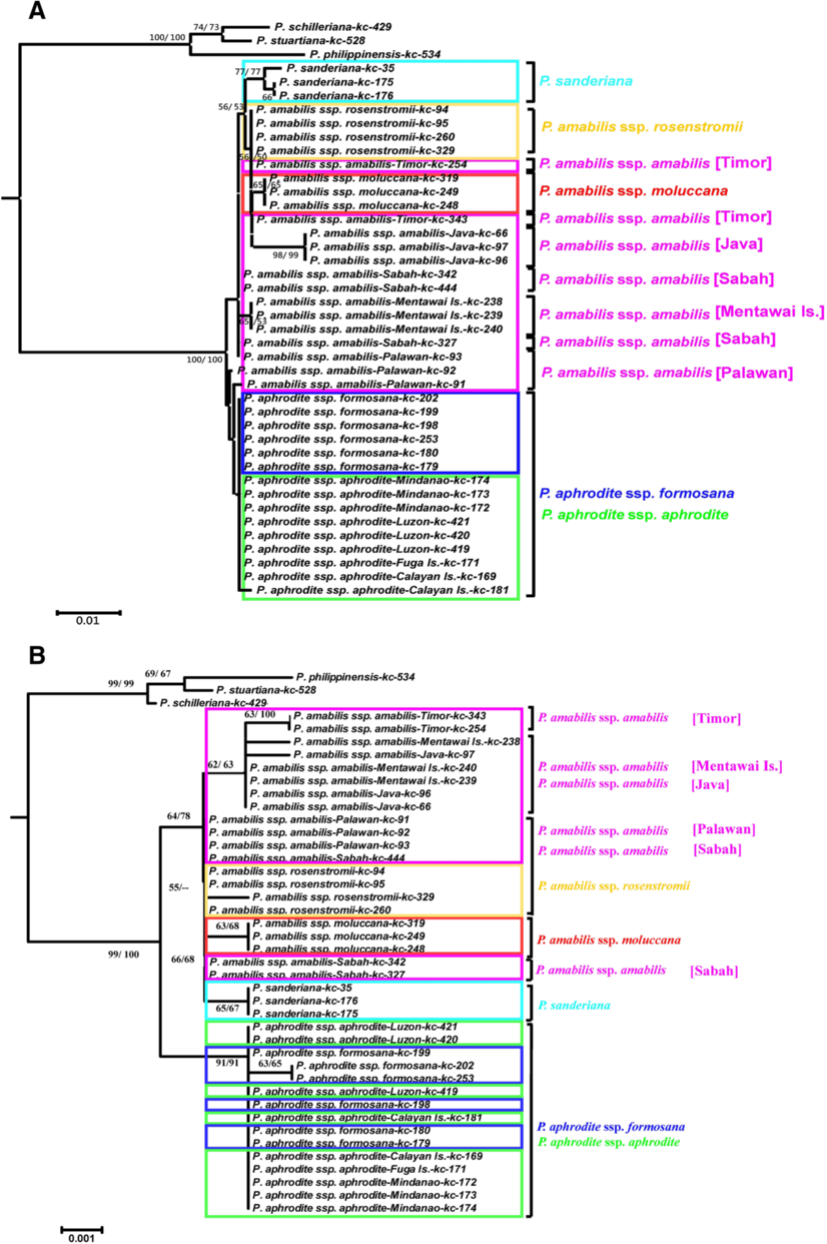
Phylogenetic relationships using Neighbor joining (NJ) and Maxinum Pasimony (MP) methods of the 39 accessions in the *P. amabilis* species complex, plus the three outgroups *P. schilleriana*, *P. stuartiana* and *P. philippinensis*, obtained from sequence comparisons of (**a**) the ITS region of nrDNA and (**b**) the cpDNA *trn*H-*psb*A spacer sequence and generated by MEGA 6.0 and Phylip 3.65. Numbers at nodes represent bootstrap values over 50 % of NJ and MP between major lineages

Genealogy was reconstructed based on the ITS1 and ITS2 and plastid DNA haplotypes of the *P. amabilis* complex, rooted at three species of the *P. schilleriana* complex, including *P. schilleriana*, *P. stuartiana*, and *P. philippinensis* (Fig. 2). In the ITS1 and ITS2 sequences, a total of 13 variable sites (nucleotide substitutions) and 7 indels were found among the species/subspecies of the *P. amabilis* complex. Based on the phylogenetic tree, the six taxa of the *P. amabilis* complex were closely related with high bootstrap supporting in both NJ and MP phylogenetic trees (Figs. 2a). *Phalaenopsis aphrodite* ssp. *aphrodite* and *P. aphrodite* ssp. *formosana* clustered together. Subspecies of *P. aphrodite* are not distinguishable from each other, whereas they were separable from other members of the *P. amabilis* complex. Subspecies of *P. amabilis*, except for the Palawan populations, and *P. sanderiana* formed a clade. Within the *P. amabilis*/*P. sanderiana* clade, two subclades were identified, rooted by *P. amabilis* ssp. *amabilis* from Palawan. The first subclades showed distinct geographic subdivision, including *P. sanderiana*, *P. amabilis* ssp. *amabilis* from Java and ssp. *moluccana* from Molucca island. Nevertheless, *P. amabilis* ssp. *amabilis* from Timor and *P. amabilis* ssp. *rosentromii* cannot be distinguished from each other. The other subclade included all *P. amabilis* ssp. *amabilis* from Mentawai Island, Sabah (Borneo) and the one from Palawan (Fig. 2a).

**Fig. 3:**
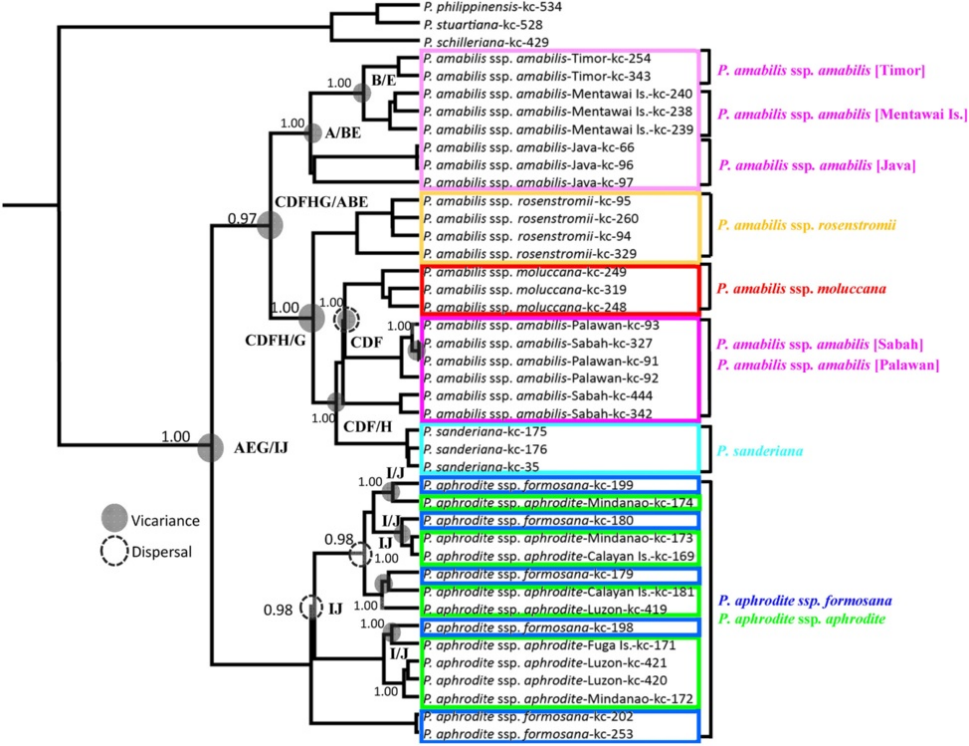
Ancestral distributions reconstructed by RASP. Phylogenetic relationships of the 39 accessions in the *P. amabilis* species complex, plus the three outgroups *P. schilleriana*, *P. stuartiana* and *P. philippinensis*, obtained from sequence comparisons of the cpDNA *trnH*-*psbA* spacer sequence and generated by BEAST. Bayesian credibility values (PP) are indicated above the branch on one of the post-burn Bayesian trees. The distribution areas of extant accessions of *P. amabilis* species are marked in capitals A–J (A: Bantam, Java, Indonesia; B: Mentawai Is., Sumatra, Indonesia; C: Brooks Point, Palawan, the Philippines; D: Sabah, Indonesia; E: East Timor; F: Celebes, Molucca Is., Indonesia; G: Kaiser Wilhelms, New Guinea; H: Mindanao, the Philippines; I: Manila, Luzon, Fuga Is., and Calayan Is. in the Philippines; J: southern Taiwan), respectively. The grey circles indicate the vicariance events and the circles with dashed line indicate the long-distance dispersal (LDD) events obtained from the RASP analysis, respectively

In the *trn*H-*psb*A spacer, 11 variable sites and 6 indels were found among the species/subspecies of the *P. amabilis* complex. The phylogeny inferred from the plastid DNA was largely consistent with those from the ITS data and provided better resolution in the geographic subdivision. The *Phalaenopsis amabilis* complex consisted of two clades with high bootstrapping supports in both NJ and MP phylogenetic trees, one for *P. amabilis* with its subspecies and *P. sanderiana* and the other for *P. aphrodite* (Figs. 2b and 3). The clade including *P. amabilis*/*P. sanderiana* consisted of two geographic groups (Fig. 2b), however, the Timor population was not closely related to *P. amabilis* ssp. *rosentromii* but was related to the Mentawei Island population (Fig. 2b)

### Divergence time estimates

Based on nuclear and plastid DNAs, tests for the molecular-clock hypothesis using Tajima’s *D* revealed no significant violations of the assumption of selective neutrality (Table 3). Based on substitution rates of 1.82 × 10^−9^ subs/site/yr for the plastid DNA spacer, the coalescence time of the *P. amabilis* complex was estimated to be 0.795 Mya, with 95 % confidence intervals (95 % CI) of 0.484–1.549 Mya (Table 5). Within the *P. amabilis* complex, the coalescence times based on the *trn*H-*psb*A spacer were estimated at 0.589 Mya (95 % CI: 0.310–1.057 Mya), 0.462 Mya (95 % CI: 0.195–0.825 Mya), and 0.138 Mya (95 % CI: 0.018–0.316 Mya) for three taxa within *P. amabilis* complex. In addition, the coalescence time of *P. amabilis* ssp. *moluccana* was tracked back to 0.069 Mya (95 % CI: 0.003–0.309 Mya) (Table 5).

**Table 5:** Results of calescence time estimations performed with BEAST 1.8.0 for the *Phalaenopsis amabilis* complex based on the *trn*H-*psb*A spacer of chloroplast DNA

	Coalescence times (Mya)					
	Substitution rate =		Substitution rate =		Substitution rate =	
	1.82 × 10^−9^ subs/site/yr		1.11 × 10^−9^ subs/site/yr		2.53 × 10^−9^ subs/site/yr	
Clade	Time	95 % CI	Time	95 % CI	Time	95 % CI
*P. amabilis* complex	0.795	0.484 ~ 1.549	0.485	0.295 ~ 0.945	1.105	0.673 ~ 2.153
*P. amabilis*	0.589	0.310 ~ 1.057	0.359	0.189 ~ 0.645	0.819	0.430 ~ 1.469
*P. aphrodite*	0.462	0.195 ~ 0.825	0.282	0.119 ~ 0.503	0.642	0.271 ~ 1.147
*P. sanderiana*	0.138	0.018 ~ 0.316	0.084	0.011 ~ 0.193	0.192	0.025 ~ 0.439
*P. amabilis* ssp. *moluccana*	0.069	0.003 ~ 0.309	0.042	0.002 ~ 0.188	0.096	0.004 ~ 0.430

Using nucleotide-substitution rates of 1.11–2.53 × 10^−9^ subs/site/yr for the plastid DNA spacer, the coalescence time of the *P. amabilis* complex was estimated at 0.485 Mya (95 % CI: 0.295–0.945 Mya) and 1.105 Mya (95 % CI: 0.673–2.153 Mya) for the plastid DNA spacer (Table 5). Likewise, the coalescence time based on the substitution rates at the *trn*H-*psb*A spacer were estimated at 0.359–0.819 Mya, 0.282–0.642 Mya, and 0.084–0.192 Mya for three taxa within the *P. amabilis* complex. Furthermore, the coalescence time of *P. amabilis* ssp. *moluccana* was tracked back to 0.042–0.096 Mya (Table 5).

### Demographic history and historical biogeography inference

**Fig. 4:**
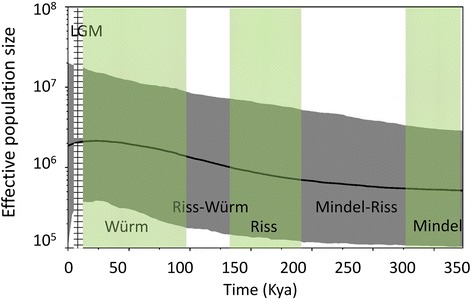
The effective population size over time for all accessions in the *P. amabilis* species complex based on the cpDNA *trn*H-*psb*A spacer using Bayesian skyline plots analyses. The Last Glacial Maximum (LGM) is green color shaded. Solid lines indicate median estimations; area between gray dash lines indicates 95 % confidence intervals

A Bayesian skyline plot was used to estimate population dynamics. Plastid DNA showed historical demographic growth followed by recent steadiness in the *P. amabilis* complex in the late LGM due to extinction of regional populations (Fig. 4). Mismatch analyses of plastid DNA sequences displayed a multimodal distribution pattern with a non-significant sum of squared deviations (SSD) statistic and the raggedness index (HRag) value under both sudden- and spatial-expansion models in the *P. amabilis* complex (Table 6). In addition, negative *Fu’s F*_*S*_ values suggested demorgraphic expansion under both sudden- and spatial-expansion model in *P. amabilis* (Table 6).

**Table 6:** Results of demographic analyses performed with DnaSP and ARLEQUIN for the *Phalaenopsis amabilis* complex based on the *trn*H-*psb*A spacer of chloroplast DNA

Group	Fu's *F*s	*SSD* ^a^	H_*Rag*_^a^	*SSD* ^b^	H_*Rag*_^b^
*trn*H-*psb*A spacer	−1.645	0.0143	0.0328	0.0153	0.0328
*P. amabilis*	−2.715*	0.0149	0.1079	0.0149	0.1079
*P. aphrodite*	−0.133	0.2814	0.3161	0.0008	0.3161
*P. sanderiana*	-----	-----	-----	-----	-----

^a^the indices under the sudden expansion model

^b^the indices under the spatial expansion model

^*^ *p* < 0.05, ^**^ *p* < 0.01

**Fig. 5:**
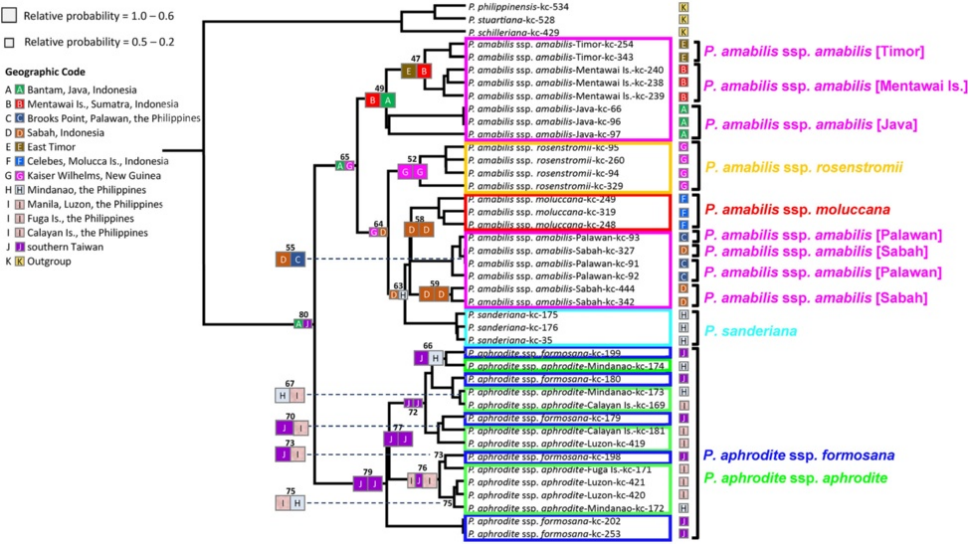
Historical biogeographical reconstruction using Lagrange on the *P. amabilis* species complex topology. Coloured squares indicate reconstructed ancestral ranges and the square size is proportional to the probability of the reconstructions (see Table 7). The geographic ranges of species are displayed at right. [left | right]: ‘left’ and ‘right’ are the ranges inherited by each descendant branch (in the printed tree, ‘left’ is the upper branch, and ‘right’ the lower branch). The distribution areas of extant accessions of *P. amabilis* species are marked in capitals A–J (A: Bantam, Java, Indonesia; B: Mentawai Is., Sumatra, Indonesia; C: Brooks Point, Palawan, the Philippines; D: Sabah, Indonesia; E: East Timor; F: Celebes, Molucca Is., Indonesia; G: Kaiser Wilhelms, New Guinea; H: Mindanao, the Philippines; I: Manila, Luzon, Fuga Is., and Calayan Is. in the Philippines; J: southern Taiwan), respectively

To infer vicariance and dispersal events in the *P. amabilis* complex, ancestral ranges were obtained by RASP analysis of plastid DNA (Fig. 3). RASP detected 17 vicariance, 36 dispersal, and 7 extinction events in the whole phylogenetic demography, suggesting a complicatedbiogeographical history in which vicariance, dispersal, and vicariance + dispersal played critical roles in shaping the current distribution in *P. amabilis* species complex. Accordingly, the extant distributions of species and subspecies of the *P. amabilis* complex may have largely been shaped by vicariance events, with relatively rare events of dispersal + vicariance or dispersal. The results supported vicariance events between *P. amabilis* and *P. aphrodite* (Fig. 3), between *P. amabilis* and *P. sanderiana*, and between *P. amabilis* ssp. *rosenstromii* and ssp. *moluccana*/ssp. *amabilis* from Sabah and Palawan/*P. sanderiana* (Fig. 3). In contrast, the analysis of plastid DNA suggested that the extant distribution of *P. aphrodite* was likely shaped by dispersal (Fig. 3). The patterns of the biogeographic reconstruction executed by Lagrange are presented in Fig. 5 and Table 7. Lagrange and RASP methods created similar patterns. However, Lagrange generally estimated in terms of relative probabilities at nodes and a better range of possible ancestral areas at nodes usually inferred a favored reconstruction. The ancestral range of *P. amabilis* complex (node 80) was estimated to have been in the Java and southern Taiwan with 0.174 of relative probability (Fig. 5 and Table 7). Likewise, The ancestral ranges of *P. aphrodite* (node 76), *P. amabilis* (node 65), and *P. sanderiana* (node 60) were likely in southern Taiwan with 0.588 of relative probability, Java / New Guinea with 0.223 of relative probability, and Mindanao with 0.956 of relative probability, respectively.

**Table 7:** The ancestral areas inferred through Lagrange. Relative probability is the fraction of the global likelihood of a split

Node	Ancestral range	-ln(L)	Relative probability
80	[A|J]	−100.1	0.174
79	[J|J]	−98.84	0.588
77	[J|J]	−98.68	0.689
76	[IJ|I]	−98.43	0.886
75	[I|H]	−98.41	0.913
73	[J|I]	−98.44	0.875
72	[J|J]	−99.22	0.401
70	[J|I]	−98.45	0.862
67	[H|I]	−98.36	0.942
66	[J|H]	−98.53	0.796
65	[A|G]	−99.81	0.223
64	[G|D]	−99.61	0.271
63	[D|H]	−99.32	0.363
59	[D|D]	−98.32	0.981
58	[D|D]	−98.82	0.596
55	[D|C]	−113.5	1.000
52	[G|G]	−98.50	0.825
49	[B|A]	−98.76	0.635
47	[E|B]	−98.76	0.638

The distribution areas of extant accessions of *P. amabilis* species are marked in capitals A–J (A: Bantam, Java, Indonesia; B: Mentawai Is., Sumatra, Indonesia; C: Brooks Point, Palawan, the Philippines; D: Sabah, Indonesia; E: East Timor; F: Celebes, Molucca Is., Indonesia; G: Kaiser Wilhelms, New Guinea; H: Mindanao, the Philippines; I: Manila, Luzon, Fuga Is., and Calayan Is. in the Philippines; J: southern Taiwan), respectively

## Discussion

### Phylogeny of the *Phalaenopsis amabilis* species complex

The taxonomy of the *Phalaenopsis amabilis* species complex is difficult to resolve [8, 9], given very few diagnosable morphological characters and occurrence of interspecific hybridisation yields numerous intermediate morphs. Nevertheless, unique biogeographical patterns with high levels of endemism on islands exist in the Sunda Shelf and the Philippine Archipelagos [6, 11], reflecting interesting but complicated divergence processes. In this study, two molecular markers provided supplementary information, with patterns in nrITS shaped by concerted evolution [19], whereas plastid DNA genes showed no evidence of genetic recombination [20]. Phylogenetic trees derived from nuclear and plastid DNAs supported the monophyly of the species complex. *P. aphrodite* ssp. *aphrodite* and *P. aphrodite* ssp. *formosana* formed a sister group to other species, but are difficult to distinguish from each other. Morphologically, *P. aphrodite* ssp. *formosana*, which lacks anthocyanin, is only slightly differentiated from *P. aphrodite* ssp. *aphrodite* [9].

*Phalaenopsis amabilis* is distinguishable from *P. aphrodite*, and both display geographic subdivision based on characteristics of the callus. The reciprocal monophyly of three subspecies of *P. amabilis*, with *P. amabilis* ssp. *moluccana* and *P. amabilis* ssp. *rosenstromii* clustering first was supported by the plastid-DNA-based tree (Fig. 2b) but not the nuclear-DNA-based tree (Fig. 2a). The systematic inconsistency between the two trees might derive from differences in the modes of inheritance, with plastid DNA and the ITS region being maternally and biparentally inherited, respectively [21, 22]. Close affinity between sister groups of *P. amabilis* and *P. sanderiana* based on plastid DNA (Fig. 2b) revealed a biogeographcial pattern subdivided by Huxley’s Line (Fig. 1) [11]. In addition, the *P. amabilis* population of Timor and two subspecies of *P. amabilis* distributed in regions east of Wallace’s Line (Fig. 1) formed a clade in the nuclear-DNA-based tree, supporting another geographic subdivision by Weber’s Line. Nevertheless, some samples from Palawan populations mixed with those of the *P. amabilis* complex in a nuclear-ITS-based tree, indicating possible paternal gene flow (Fig. 1). This result suggests long-distance pollen dispersal in *P. amabilis*. Such dispersal would usually occur with the aid of large pollinators, such as carpenter bees (*Xylocopa*) [9], which are capable of flying [23].

Morphologically, the callus structure of *P. sanderiana* can be distinguished from those of *P. amabilis* and *P. aphrodite* [9]. According to phylogenetic trees derived from nuclear and plastid DNAs*, P. sanderiana* alone formed a clade, nested in *P. amabilis*, making the latter species paraphyletic.

### Biogeography and evolutionary trends

Gene genealogies were used to extract data on historical gene flow [24], hybridisation [25] and divergence between lineages [26–29]. According to the phylogenies based on nuclear (Fig. 2) and plastid DNA (Fig. 3), *P. aphrodite* ssp. *aphrodite* and ssp. *formosana* distributed in the Philippines and Taiwan is a sister species to the rest of the *P. amabilis* complex. *P. aphrodite* ssp. *formosana* is geographically restricted to Taiwan. This subspecies represented the northernmost limitation in distribution for *Phalaenopsis*, while *P. aphrodite* is distributed throughout the Philippines [8]. In addition, the Philippines are the centre of diversity for species of this group of *Phalaenopsis* spp. (*P. amabilis* complex and *P. schilleriana* complex) [30], as all members of the section *Phalaenopsis*, except for *P. amabilis*, were found in the Philippines [8, 9]. In addition, several old islands of the Philippines, including Palawan, Mindoro and Zamboanga, were separated from the Eurasian Plate when the crust of the plate hit Borneo approximately 5–10 Mya [3–5], providing a refuge for the diverging species.

*Phalaenopsis aphrodite* is absent in Palawan and southern Mindanao [8, 9]. These two islands were interconnected during the glacial periods [6] (Fig. 1), providing opportunities for population expansion. Furthermore, *P. aphrodite* probably dispersed into Taiwan during the glacial maxima, as the Taiwan-Luzon-Mindoro belt was formed in the late Eocene [4]. Phylogenetically, *P. aphrodite* ssp. *formosana* and *P. aphrodite* are very similar based on the nuclear ITS and the chloroplast *trn*H-*psb*A spacer. The coalescence of *P. aphrodite* can be traced to approximately 0.535 Mya (95 % CI: 0.257–0.953 Mya), ranging from 0.312 to 0.758 Mya (plastid *trn*H-*psb*A) during the Pleistocene glaciations. The Philippine Archipelagos, except for Palawan, formed the super island [31] and were periodically linked and unlinked to the Sunda Shelf by the land bridge between Mindoro, Palawan, and Borneo. The most recent link likely formed approximately 0.01–1.8 Mya as a result of lowered Pleistocene sea levels [6, 32] and *P. aphrodite* probably dispersed among Mindoro, Palawan, Borneo, and Taiwan. This geographic disjunction likely contributed to the vicariance between the Philippine Archipelagos and continental Asia.

Geographically, *P. amabilis* is separated from *P. amabilis* ssp. *moluccana* and *P. amabilis* ssp. *rosenstromii* by Wallace’s Line. There is divergence between floral and faunal species in the regions west and east of Wallace’s Line [6, 33, 34]. Biogeographical subdivision also occurred between *P. amabilis* ssp. *moluccana* (Sulawesi) and *P. amabilis* ssp. *rosenstromii* (New Guinea/Australia). The coalescence of *P. amabilis* can be dated to 0.589 Mya (95 % CI: 0.310–1.057 Mya) by the plastid *trn*H-*psb*A, likely in association with the Würm glaciation. This finding is in agreement with the phylogeographical break described by Weber [11]. The current biota in this geographic area therefore show evidence of vicariace from the Sunda Shelf west of New Guinea/Australia [33, 34].

Inconsistent phylogenetic demographies between nuclear and plastid markers indicate historical introgression/hybridisation [35, 36] between *P. amabilis* and *P. aphrodite* (Fig. 2) may due to recombination by paternal gene flow . The *P. amabilis* distributed in Palawan is closely related to the *P. amabilis* distributed in Borneo based on maternal molecular data and historical connection by land bridges [37, 38] between the Sunda Shelf and the Philippine Archipelagos, however, *P. amabilis* was paraphyletic according to the nuclear ITS, with one major clade distributed in Palawan and one haplotype clustering with *P. aphrodite*. The coalescence time based on plastid data was less than 0.10 Mya between the Palawan and Borneo populations, indicating a very recent split. That result is consistent with the topophysiographic data [39].

*Phalaenopsis sanderiana* forms a clade (Figs. 2, 3 and 5) linked to *P. amabilis* of Borneo/Palawan. Geographically, *P. sanderiana* is exclusively found in southern Mindanao [8, 9]. Several islands between southern Mindanao and Borneo may have formed a land bridge during the glacial maxima, providing opportunities for dispersal from Borneo to southern Mindanao [6, 33, 34].

RASP analyses also suggested that the current disjunct distribution of the *P. amabilis* complex might reflect vicariant relicts of an ancestral population (Fig. 3). This finding concurs with findings for other disjunctions at the Sunda Shelf and Philippine Archipelagos or Sunda Shelf and New Guinea/Australia [33, 34]. In addition, several vicariance, dispersal, and vicariance + dispersal events detected using RASP (Fig. 3) revealed geographic isolation among populations/taxa during the Pleistocene, likely as a result of the landbridge submergence [38, 40, 41].

### Conservation genetics of *P. amabilis* complex

*Phalaenopsis amabilis* complex represents a group of epiphytes of typical evergreen forests throughout tropical Asia and the larger islands of the Pacific Ocean; the whole region was the ancient Sunderland and Great Sunda Island during the Pleistocene glaciation [6]. As epiphytic species widespread in tropical Asia, the *P. amabilis* complex is expected to have had a large ancestral population. In this study, population growth was detected with sudden- and spatial-mismatch distribution models for three taxa and the species complex based on the Bayesian skyline plots using BEAST program for species. At plastid marker, a continuous demographic expansion from Mindel to Würm pleistocene period of the *P. amabilis* complex was detected, followed by subsequent reversed population slowdown after LGM (Fig. 4). Climate changes in the Quaternary that triggered isolation and local extinction based on 7 extinction events detected by RASP (thus promoting speciation) may explain the increased differentiation rates during that period [42, 43]. During the Pleistocene, geographic fluctuations in the Sunda Shelf, Philippine Archipelagos, and New Guinea/Australia resulted in population fragmentation. At the same, colonization via long distance dispersal also occurred. The ancestral population of the *P. amabilis* complex was likely to split into *P. aphrodite*, *P. amabilis*, and *P. sanderiana* by geological vicariance associated with Huxley’s Line [44] and Wallace’s Line [11].

At the species level, the nucleotide diversity of nuclear DNA (*P. amabilis*: 0.0054, *P. aphrodite*: 0.0005, *P. sanderiana*: 0.0028) and cpDNA (*P. amabilis*: 0.0012, *P. aphrodite*: 0.0000, *P. sanderiana*: 0.0029) was low (Table 4) compared to other flowering plants from the same geographic region, such as *Kandelia candel* (nucleotide diversity *θ* = 0.02652, Chiang et al. [45]), *Ceriops tagal* (nucleotide diversity *π* = 0.01703, Liao et al. [46]), *Bruguiera gymnorrhiza* (*π* = 0.08, Urashi et al. [47]). As an epiphyte growing in vulnerable habitats across a wide distribution, all local populations tend to be small, strengthening the effects of genetic drift [48]. Therefore, taxa of the *P. amabilis* complex tend to have low genetic diversity at the intraspecific level in both nuclear and plastid DNAs and populations are fixed at a single haplotype (Table 4).

Moreover, the nucleotide diversity of other sympatrically distributed species and the *P. amabilis* complex reflects the effects of a common geological history, likely including the Quaternary glacial circle [38, 40, 49, 50]. At the population and species levels, the combination of low genetic diversity and high levels of genetic differentiation in the *P. amabilis* complex indicate that populations of this species were isolated from each other and experienced either extreme bottlenecks [29, 51–53] or founder effects [54, 55]. Furthermore, the unique interactions between orchids and pollinators [56] and the habitat preferences [57] strengthened the genetic differentiation among populations/species. Given such genetic background, most species, apparently, face threats of human exploitation of natural forests that inevitably led to shrinkage of populations of the species complex. Conservation of these evolving species/subspecies is therefore urgent and required.

## Conclusions

In summary, variations in nuclear and plastid DNA revealed that the disjunct distribution of the *P. amabilis* complex likely resulted from vicariance or vicariance + dispersal in the middle–late Pleistocene. Due to the submergence of the Sunda Shelf, Philippine Archipelagos, and New Guinea/Australia, *P. aphrodite* was separated from other members of the *P. amabilis* complex. Within *P. aphrodite*, the admixture of populations made subspecies indistinguishable, suggesting a history of long-distance dispersal. In contrast, the species and subspecies of *P. amabilis* with disjunct distributions were differentiated due to vicariance. After divergence, the species complex experienced isolation and *in situ* range expansion as well as population growth during subsequent climatic oscillations in the Würm glacial period. Population slowdown following the end of the LGM may have occurred by an extinction of local or island populations owing to a reduction in suitable habitats and formed the contemporary pattern of geographic distribution.

## Methods

### Plant materials

Thirty-nine accessions of the *P. amabilis* complex were obtained from 13 different populations, and three species of the *P. schilleriana* complex were used as outgroups (Table 2) [37, 58]. Four individuals were sampled from each accession. Leaf materials were taken from living plants. All samples examined in this study were collected from wild populations and cultivated at the green house of the Kaohsiung District Agricultural Improvement Station (KDAIS) in Taiwan, and the voucher specimens were deposited at the herbarium of the National Museum of Natural Science, Taiwan (TNM). The geographic distributions and flower photos of species of this complex are shown in Fig. 1.

### DNA extraction, PCR amplification and sequencing

Using the cetyltrimethylammonium bromide (CTAB) method [59], total DNA was extracted from fresh etiolated leaves. Ethanol-precipitated DNA was dissolved in TE (Tris-EDTA) buffer and stored at −20 °C. Qiagen (Valencia, CA, USA) columns were used to clean the DNA samples that were difficult by amplify by PCR. Approximate DNA yields were determined using a spectrophotometer (model U-2001, Hitachi).

Amplification protocols were as follows: each 50-μL reaction contained 40 mM Tricine-KOH (pH 8.7), 15 mM KOAc, 3.5 mM Mg(OAc)_2_, 3.75 μg ml^−1^ BSA, 0.005 % Tween 20, 0.005 % Nonidet-P40, four dNTPs (0.2 mM each), primers (0.5 μM each), 2.5 units of Advantage 2 DNA polymerase (Clontech, USA), 10 ng of genomic DNA, and 50 μL of mineral oil. Amplification reactions were completed in a dry-block with two-step thermal cycles (Biometra, Germany). Two primers designed to amplify *Phalaenopsis* ITS and the PCR conditions were described in Tsai et al. [58], and the primers and conditions for the *trn*H-*psb*A spacer were described in Taberlet et al. [60]. The PCR conditions for the *trn*H*-psb*A spacer were as follows: incubation at 94 °C for 3 min, 10 cycles of denaturation at 94 °C for 45 s, annealing at 54 °C for 30 s, and extension at 72 °C for one min, 30 cycles of denaturation at 94 °C for 45 s, annealing at 52 °C for 30 s, extension at 72 °C for one min, and a final extension for five min at 72 °C. PCR products were stained with 0.5 μg ml^−1^ ethidium bromide, detected by agarose-gel electrophoresis (1.0 % w v^−1^, in TBE), and photographed under UV light.

All PCR products of different DNA fragments from the plant material were recovered by glassmilk (BIO 101, California) and sequenced directly by the dideoxy chain-termination method, using an ABI3730 automated sequencer with a Bigdye^TM^ Terminator Cycle Sequencing Ready Reaction Kit (PE Biosystems, California). The sequencing primers were the same as those used for PCR. These reactions were performed according to the recommendations of the manufacturers.

### Sequence alignment and genetic-diversity analysis

The sequences of the nrITS region and *trn*H-*psb*A spacer were deposited in GenBank under accession numbers of AY391515-AY391553 and FJ460366-FJ460407, respectively. The sequences were aligned using the MUSCLE multiple-alignment program in MEGA 6.0 [61]. Genetic diversity was evaluated based on indices of haplotype diversity [62] and nucleotide diversity [62] using DnaSP 5.10.1 [63].

### Phylogenetic reconstruction

Insertion/deletions (indels) were coded as missing data. For phylogenetic analyses, the three outgroups were included to determine whether all recovered haplotypes formed a monophyletic lineage. Phylogenetic relationships were inferred using NJ in MEGA 6.0 [61] and MP in PHYLIP 3.65 [64], with the best-fitting Tamura 3-paramenter model (T92) using a discrete Gamma distribution (+G) for the nrITS region (Additional file 2: Table S2) and Tamura 3-paramenter model (T92) [65] for the *trn*H-*psb*A spacer (Additional file 3: Table S3) selected by a model test using MEGA 6.0 [61], with indels treated as missing data. Bootstrapping (1000 replicates) was carried out to estimate the support for NJ and MP topology [66, 67]. The NJ phylogenetic tree was drawn with bootstrapping values using the MEGA 6.0 and combined the bootstrapping information from MP phylogenetic tree.

### Divergence time estimation

To avoid the noise of introgression and recombination due to paternal gene flow, we estimated the divergence time and historical demographic evaluation exclusively based on non-coding cpDNA regions. For estimating divergence time, a Bayesian estimate of group delimitation and the ages of TMRCA of the moth orchid clades on each node were inferred using Yule model methods in BEAST 1.8.0 [68], with the best-fitting HKY model selected by a model test using MEGA 6.0 [61], and indels treated as missing data. For Bayesian analysis, four parallel runs and four Markov Chain Monte Carlo (MCMC) chains, each with a different starting seed, were run for 100,000,000 generations. A tree was sampled every 10,000 generations after a burn-in period of 10,000,000 generations, after which the standard deviation of the split frequencies was below 0.01, per the suggestion in the manual. BEAST 1.8.0 was used to reconstruct the gene trees and to simultaneously estimate divergence between and within clades. As the taxon-specific substitution rate had not previously been calibrated for non-coding cpDNA regions of the *P. amabilis* complex, we used the synonymous (*Ks*) substitution rates of 0.36 ± 0.14 per site for cpDNA coding regions between *P. aphrodite* and grasses [69] as the reference, based on the coalescence time of 99 Mya, which is the age of the oldest known Asparagales [70]. We estimated a substitution rate of 1.82 × 10^−9^ subs/site/yr and set 1.11 × 10^−9^ subs/site/yr as the lower limit and 2.53 × 10^−9^ subs/site/yr as the upper limit for the nucleotide-substitution rate of the cpDNA spacer in our analyses. All summary statistics of the output values were summarised using Tracer 1.5 [71], and both log and tree files of the last four runs were combined using LogCombiner 1.6.1 [72]. TreeAnnotator 1.6.1 [72] and FigTree 1.3.1 [73] were used to summarise and display the sampled trees, respectively.

### Historical demographic evaluation

To examine the demographic history of *P. amabilis* complex, nucleotide sequences were tested using Tajima’s D test of neutrality [74] and Fu's *Fs* statistics [75], which are powerful for detecting population growth [76], using DnaSP 5.10.1. Mismatch analysis was performed to evaluate the range expansions of the *P. amabilis* complex under the sudden-expansion model using Arlequin var. 3.5.1.2 [77] and was compared to Poisson distributions. The SSD between the expected and observed mismatch distributions and *P*-values were calculated. The HRag and its significance for observed distributions were used to compute the smoothness of the mismatch distributions [78]. Low raggedness is typical of recently non-stationary, expanding populations.

To investigate the historical demographics, a coalescence approach with a Bayesian skyline plot was used to evaluate the dynamic history of the *P. amabilis* complex using BEAST 1.8.0. The best-fitting substitution model, substitution rate, and general parameter setting followed the phylogenetic reconstruction using BEAST described above. All summary statistics of the output values and the Bayesian skyline plot were generated using Tracer 1.5 [71], and the log and tree files of the last four runs were combined using LogCombiner 1.6.1 [72].

### Biogeographic inference using RASP and Lagrange

To infer the biogeographic history of the *P. amabilis* complex and thereby distinguish the effects of vicariance from dispersal, we condcuted two different approaches. First, we used the Statistical dispersal–vicariance analysis (S-DIVA) and Bayesian binary MCMC (BBM) analysis, performed in RASP 2.0 [79]. RASP is one of the most widely used methods for inferring biogeographic histories, and distingushing vicariance from dispersal. Second, we used Lagrange v. 20130526 [80] to test the complex models of directional biogeographic expansion and inferred the geographic origin of the clade based on a dispersal-extinction-cladogenesis (DEC) model employing a maximum likelihood framework for testing models of geographic range evolution. To clarify the vicariance and dispersal events, the geographic distributions of the *P. amabilis* complex were mapped onto 10 areas across islands: (A) Bantam, Java, Indonesia; (B) Mentawai Is., Sumatra, Indonesia; (C) Brooks Point, Palawan, the Philippines; (D) Sabah, Boeneo, Indonesia; (E) East Timor; (F) Celebes, Molucca Is., Indonesia; (G) Kaiser Wilhelms, New Guinea; (H) Mindanao, the Philippines; (I) Manila, Luzon, Fuga Is., and Calayan Is. in the Philippines; and (J) southern Taiwan. The tree topologies constructed with BEAST and 10,000 trees were used in S-DIVA and Lagrange analysis. At each node, the number of maximal areas endorsed in ancestral distributions was set as 3 and other parameters were automatically implemmented in S-DIVA. The range matrix defining the region from each specimen of moth orchids and a phylogenetic tree was uploaded to the Lagrange configurator [80], biogeographic models were defined as allowing for two potential ancestral regions per node in the Lagrange analyses.

## Availability of supporting data

The data used to perform the phylogenetic analyses is available at Dryad [http://dx.doi.org/10.5061/dryad.f8j12].

## Additional files

Additional file 1: Table S1. One part of the cpDNA *trn*H-*psb*A spacer sequence alignment. Three different types of repeat sequences were found and are marked by gray regions. Additional file 2: Table S2. The model test for *Phalaenopsis amabilis* species complex based on the nrITS region using MEGA 6.0. Additional file 3: Table S3. The model test for *Phalaenopsis amabilis* species complex based on the plastid noncoding region using MEGA 6.0.

## Abbreviations

NJ: Neighbor joining
MP: Maxinum parsimony
BEAST: Bayesian evolutionary analysis sampling trees
RASP: Reconstruct Ancestral State in Phylogenies
LGM: Last glacial maximum
Mya: Million years ago
KDAIS: Kaohsiung District Agricultural Improvement Station
TNM: National Museum of Natural Science, Taiwan
CTAB: Cetyltrimethylammonium bromide
T92: Tamura 3-paramenter model
MCMC: Markov Chain Monte Carlo
SSD: Sum of squared deviations
HRag: The raggedness index
S-DIVA: Statistical dispersal–vicariance analysis
BBM: Bayesian binary MCMC
DEC: dispersal-extinction-cladogenesis
