# Supplementary material for: Biogeography of the Phalaenopsis amabilis species complex inferred from nuclear and plastid DNAs
Source: BMC Plant Biol. 2015 Aug 16;15:202. doi: 10.1186/s12870-015-0560-z (PMC4537552; doi:10.1186/s12870-015-0560-z)
Supplement: Additional file 1: Table S1. — One part of the cpDNA trnH-psbA spacer sequence alignment. Three different types of repeat sequences were found and are marked by gray regions. [file 12870_2015_560_MOESM1_ESM.pdf]

Additional file 1: Table S1

```
                                      120        130        140        150        160        170        180        190        200        210
P. amabilis-kc-66                     TTTTTGAAGA TAGCAATCCC CCAATATCTT GTTCTTAGA- ---------- --------GC AAGATATTGG GGGATTGCTA TCTTGTTGAG CCTTTTGCTT
P. amabilis-kc-96                     .......... .......... .......... .........- ---------- --------.. .......... .......... .......... ..........
P. amabilis-kc-97                     .......... .......... .......... .........- ---------- --------.. .......... .......... .......... ..........
P. amabilis-kc-238                    .......... .......... .......... .........- ---------- --------.. .......... .......... .......... ..........
P. amabilis-kc-239                    .......... .......... .......... .........- ---------- --------.. .......... .......... .......... ..........
P. amabilis-kc-240                    .......... .......... .......... .........- ---------- --------.. .......... .......... .......... ..........
P. amabilis-kc-91                     .......... .......... .......... .........- ---------- --------.. .......... .......... .......... ..........
P. amabilis-kc-92                     .......... .......... .......... .........- ---------- --------.. .......... .......... .......... ..........
P. amabilis-kc-93                     ........A. .......... .......... .........- ---------- --------.. .......... .......... .......... ..........
P. amabilis-kc-327                    .......... .......... .......... .........- ---------- --------.. .......... .......... .......... ..........
P. amabilis-kc-342                    .......... .......... .......... .........- ---------- --------.. .......... .......... .......... ..........
P. amabilis-kc-444                    .......... .......... .......... .........- ---------- --------.. .......... .......... .......... ..........
P. amabilis-kc-254                    .......... .......... .......... .........G CAATATCTTG TTCTTAGA.. .......... .......... .......... ..........
P. amabilis-kc-343                    .......... .......... .......... .........G CAATATCTTG TTCTTAGA.. .......... .......... .......... ..........
P. amabilis ssp. moluccana-kc-248     ....G..... .......... .......... .........- ---------- --------.. .......... .......... .......... ..........
P. amabilis ssp. moluccana-kc-249     ....G..... .......... .......... .........- ---------- --------.. .......... .......... .......... ..........
P. amabilis ssp. moluccana-kc-319     ....G..... .......... .......... .........- ---------- --------.. .......... .......... .......... ..........
P. amabilis ssp. rosenstromii-kc-94   .......... .......... .......... .........- ---------- --------.. .......... .......... .......... ..........
P. amabilis ssp. rosenstromii-kc-95   .......... .......... .......... .........- ---------- --------.. .......... .......... .......... ..........
P. amabilis ssp. rosenstromii-kc-260  .......... .......... .......... .........- ---------- --------.. .......... .......... .......... ..........
P. amabilis ssp. rosenstromii-kc-329  .......... .......... .......... .........- ---------- --------.. .......... .......... .......... ..........
P. aphrodite-kc-172                   .......... .......... .......... .C...A...- ---------- --------A. .......... .......... .......... ..........
P. aphrodite-kc-173                   .......... .......... .......... .C...A...- ---------- --------A. .......... .......... .......... ..........
P. aphrodite-kc-174                   .......... .......... .......... .C...A...- ---------- --------A. .......... .......... .......... ..........
P. aphrodite-kc-419                   .......... .......... .......... .C...A...- ---------- --------A. .......... .......... .......... ..........
P. aphrodite-kc-420                   .......... .......... .......... .C...A...- ---------- --------A. .......... .......... .......... ..........
P. aphrodite-kc-421                   .......... .......... .......... .C...A...- ---------- --------A. .......... .......... .......... ..........
P. aphrodite-kc-171                   .......... .......... .......... .C...A...- ---------- --------A. .......... .......... .......... ..........
P. aphrodite-kc-169                   .......... .......... .......... .C...A...- ---------- --------A. .......... .......... .......... ..........
P. aphrodite-kc-181                   .......... .......... .......... .C...A...- ---------- --------A. .......... .......... .......... ..........
P. aphrodite ssp. formosana-kc-179    .......... .......... .......... .C...A...- ---------- --------A. .......... .......... .......... ..........
P. aphrodite ssp. formosana-kc-180    .......... .......... .......... .C...A...- ---------- --------A. .......... .......... .......... ..........
P. aphrodite ssp. formosana-kc-198    .......... .......... .......... .C...A...- ---------- --------A. .......... .......... .......... ..........
P. aphrodite ssp. formosana-kc-199    .......... .......... .......... .C...A...- ---------- --------A. .......... .......... .......... ..........
P. aphrodite ssp. formosana-kc-202    .......... .......... .......... .C...A...- ---------- --------A. .......... .......... .......... ..........
P. aphrodite ssp. formosana-kc-253    .......... .......... .......... .C...A...- ---------- --------A. .......... .......... .......... ..........
P. sanderiana-kc-35                   .......... .......... .......... .........- ---------- --------.. .......... .......... .......... ..........
P. sanderiana-kc-175                  .......... .......... .......... .........- ---------- --------.. .......... .......... .......... ..........
P. sanderiana-kc-176                  .......... .......... .......... .........- ---------- --------.. .......... .......... .......... ..........
```

```
                                      220        230        240        250        260        270        280        290        300        310
P. amabilis-kc-66                     CTCTATTTTC GTTCTTTATC ATAAAAGTTT TCCCCCGCCA ATGAATGAAA TGAA------ ---TGAAATG AATGATA--- ------AGTG CCTAGGTGAA
P. amabilis-kc-96                     .......... .......... .......... .......... .......... ....------ ---....... .......--- ------.... ..........
P. amabilis-kc-97                     .......... .......... .......... .......... .......... ....------ ---....... .....A.TGA ATGATC.... ..........
P. amabilis-kc-238                    .......... .......... .......... .......... .......... ....------ ---....... .......--- ------.... ..........
P. amabilis-kc-239                    .......... .......... .......... .......... .......... ....------ ---....... .......--- ------.... ..........
P. amabilis-kc-240                    .......... .......... .......... .......... .......... ....------ ---....... .......--- ------.... ..........
P. amabilis-kc-91                     .......... .......... .......... .......... .......... ....------ ---....... .......--- ------.... ..........
P. amabilis-kc-92                     .......... .......... .......... .......... .......... ....------ ---....... .......--- ------.... ..........
P. amabilis-kc-93                     .......... .......... .......... .......... .......... ....------ ---....... .......--- ------.... ..........
P. amabilis-kc-327                    .......... .......... .......... .......... .......... ....------ ---....... .......--- ------.... ..........
P. amabilis-kc-342                    .......... .......... .......... .......... .....----- ---------- ---....... .......--- ------.... ..........
P. amabilis-kc-444                    .......... .......... .......... .......... .......... ....------ ---....... .......--- ------.... ..........
P. amabilis-kc-254                    .......... .......... ..C....... .......... .......... ....------ ---....... .......--- ------.... ..........
P. amabilis-kc-343                    .......... .......... ..C....... .......... .......... ....------ ---....... .......--- ------.... ..........
P. amabilis ssp. moluccana-kc-248     .......... .......... .......... .......... .......... ....------ ---....... .......--- ------.... ..........
P. amabilis ssp. moluccana-kc-249     .......... .......... .......... .......... .......... ....------ ---....... .......--- ------.... ..........
P. amabilis ssp. moluccana-kc-319     .......... .......... .......... .......... .......... ....------ ---....... .......--- ------.... ..........
P. amabilis ssp. rosenstromii-kc-94   .......... .......... .......... .......... .......... ....TGAAAT GAA....... .......--- ------.... ..........
P. amabilis ssp. rosenstromii-kc-95   .......... .......... .......... .......... .......... ....TGAAAT GAA....... .......--- ------.... ..........
P. amabilis ssp. rosenstromii-kc-260  .......... .......... .......... .......... .......... ....TGAAAT GAA....... .......--- ------.... ..........
P. amabilis ssp. rosenstromii-kc-329  .......... .......... .......... .......... .......... ....TGAAAT GAA....... .......--- ------.... ..........
P. aphrodite-kc-172                   .......... .......... .......... .......... .......... ....------ ---...---- -----..--- ------.... ..........
P. aphrodite-kc-173                   .......... .......... .......... .......... .......... ....------ ---...---- -----..--- ------.... ..........
P. aphrodite-kc-174                   .......... .......... .......... .......... .......... ....------ ---...---- -----..--- ------.... ..........
P. aphrodite-kc-419                   .......... .......... .......... .......... .......... ....------ ---....... .......--- ------.... ..........
P. aphrodite-kc-420                   .......... .......... .......... .......... .......... ....------ ---....... .......--- ------.... ..........
P. aphrodite-kc-421                   .......... .......... .......... .......... .......... ....------ ---....... .......--- ------.... ..........
P. aphrodite-kc-171                   .......... .......... .......... .......... .......... ....------ ---...---- -----..--- ------.... ..........
P. aphrodite-kc-169                   .......... .......... .......... .......... .......... ....------ ---...---- -----..--- ------.... ..........
P. aphrodite-kc-181                   .......... .......... .......... .......... .......... ....------ ---....... .......--- ------.... ..........
P. aphrodite ssp. formosana-kc-179    .......... .......... .......... .......... .......... ....------ ---....... .......--- ------.... ..........
P. aphrodite ssp. formosana-kc-180    .......... .......... .......... .......... .......... ....------ ---....... .......--- ------.... ..........
P. aphrodite ssp. formosana-kc-198    .......... .......... .......... .......... .......... ....------ ---....... .......--- ------.... ..........
P. aphrodite ssp. formosana-kc-199    .......... .......... .......... .......... .......... ....------ ---....... .......--- ------.... ..........
P. aphrodite ssp. formosana-kc-202    .......... .......... .......... .......... .......... ....------ ---....... .......--- ------.... ..........
P. aphrodite ssp. formosana-kc-253    .......... .......... .......... .......... .......... ....------ ---....... .......--- ------.... ..........
P. sanderiana-kc-35                   .......... .......... .......... .......... .......... ....------ ---....... .......--- ------.... ..........
P. sanderiana-kc-175                  .......... .......... .......... .......... .......... ....------ ---....... .......--- ------.... ..........
P. sanderiana-kc-176                  .......... .......... .......... .......... .......... ....------ ---....... .......--- ------.... ..........
```
